# A Flexible Portable Glucose Sensor Based on Hierarchical Arrays of Au@Cu(OH)_2_ Nanograss

**DOI:** 10.3390/s19225055

**Published:** 2019-11-19

**Authors:** Min Jiang, Peng Sun, Jie Zhao, Lihua Huo, Guofeng Cui

**Affiliations:** 1 School of Mechanical and Automotive Engineering, South China University of Technology, Guangzhou 510640, China; mejiangmin@mail.scut.edu.cn; 2 Key Laboratory for Polymeric Composite & Functional Materials of Ministry of Education, School of Chemistry, Sun Yat-sen University, Guangzhou 510275, China; sunp25@mail2.sysu.edu.cn; 3 MOE Laboratory of Bioinorganic and Synthetic Chemistry, The Key Lab of Low-Carbon Chemistry and Energy Conservation of Guangdong Province, School of Chemistry, Sun Yat-sen University, 135, Xingang West Road, Guangzhou 510275, China; 4 Key Laboratory of Functional Inorganic Material Chemistry, Ministry of Education, School of Chemistry and Materials Science, Heilongjiang University, Harbin 150080, China

**Keywords:** Cu(OH)_2_ nanograss, Au nanoparticles, glucose detection, flexible electrochemical biosensor

## Abstract

Flexible physiological medical devices have gradually spread to the lives of people, especially the elderly. Here, a flexible integrated sensor based on Au nanoparticle modified copper hydroxide nanograss arrays on flexible carbon fiber cloth (Au@Cu(OH)_2_/CFC) is fabricated by a facile electrochemical method. The sensor possesses ultrahigh sensitivity of 7.35 mA mM^−1^ cm^−2^ in the linear concentration range of 0.10 to 3.30 mM and an ultralow detection limit down to 26.97 nM. The fantastic sensing properties can be ascribed to the collective effect of the superior electrochemical catalytic activity of nanograss arrays with dramatically enhanced electrochemically active surface area as well as mass transfer ability when modified with Au and intimate contact between the active material (Au@Cu(OH)_2_) and current collector (CFC), concurrently supplying good conductivity for electron/ion transport during glucose biosensing. Furthermore, the device also exhibits excellent anti-interference and stability for glucose detection. Owing to the distinguished performances, the novel sensor shows extreme reliability for practical glucose testing in human serum and juice samples. Significantly, these unique properties and the soft structure of silk fabric can provide a promising structure design for a flexible micro-device and a great potential material candidate of electrochemical glucose sensor.

## 1. Introduction

Diabetes mellitus, also known as the disease of affluence, is caused by both genetic and environmental factors. Currently, there is no radical cure for diabetes, but it can be controlled by various treatments. It will cause many serious complications when not diagnosed and treated in time [1]. One effective way to prevent such dire consequences is to monitor blood sugar levels accurately on a daily basis. Therefore, a flexible glucose sensor with high reliability and portability is particularly important [2,3]. However, most studies have focused on the preparation and application of flexible glucose sensor materials [4,5,6,7] and integrating flexible non-enzymatic glucose sensors has rarely been reported before.

For a flexible sensor, it is equally important to fabricate a flexible electrode material and a flexible substrate such that the final sensor can be applied to a curved surface, e.g., a surface in the human body. In this aspect, carbon fiber cloth is a very ideal electrochemical flexible substrate, which has excellent electrical conductivity for deposition of active substances and soft structure of silk fabric to meet wearable needs. In addition, its macroporous structure provides a fast transport path for ions and accelerates the diffusion of electrolytes in the electrode material [8]. More importantly, research shows that electrochemical material electrodes using carbon fiber cloth as a substrate have a larger electrochemically active surface area and higher utilization of active materials, compared to applying other common substrates [9,10]. Based on the above, this paper selects and uses carbon fiber cloth as the substrate to make flexible glucose sensors.

Enzyme glucose sensors have matured over three generations, but their own fatal shortcomings, such as poor stability and high cost [11,12], have led to extensive research on non-enzyme glucose sensors [13,14], for which non-enzymatic catalytic material is a most vital factor. Because the microstructures of catalytic materials, including surface area, porosity, special structure, etc., have close contact with their biocatalytic activity [15,16], various nanostructured catalytic materials for non-enzyme glucose sensors have been widely studied, such as nanoporous metal (Au, Pt, Cu, etc. [17,18,19]) and metal oxides/hydroxides (CuO, Ni(OH)_2_, Co_3_O_4_, etc. [20,21,22,23]) and nanocomposites (Pt-CuO, ZnO-CuO, Co_3_O_4_-PbO_2_, etc. [9,24,25]). Among them, the nanocomposites of noble metal with the copper oxides are excellent materials for non-enzymatic glucose sensors, due to it possessing the properties of stable, low cost and high catalytic activity of copper oxides and its catalytic performance being further enhanced by noble metal [3,24,26,27,28]. For copper-based non-enzyme glucose sensors, the possible electrocatalytic mechanism of glucose oxidation has been attributed to the mediation by Cu(OH)_2_/CuOOH [29,30]. Thus, it can be better to fabricate copper hydroxide electrode materials instead of copper oxides for glucose detection. However, there are only a few works on copper hydroxide electrode research and they have also demonstrated improved catalytic performance for glucose [30,31,32]. Even more regrettably, there is no nanocomposite material of noble metal–copper hydroxide. In addition, the noble metal–copper oxides sensing catalyst is fabricated by a one step chemical process and immobilized on hard substrates such as the screen printed electrodes and glassy carbon electrodes [24,27,28]. But the chemical syntheses are usually energy-consuming and could result in impurities that hinder glucose sensing [6]. In contrast, the electrochemical synthesis provides a facile method to fabricate the oxides and hydroxides of copper and deposit noble metal on the flexible substrates [20,33,34].

In this paper, we produced flexible electrodes on carbon fiber cloth (CFC) by a facile electrochemical method, such as the working electrode of Au nanoparticle modified copper hydroxide nanograss arrays (Au@Cu(OH)_2_/CFC), counter electrode of Pt/CFC and reference electrode of Ag/AgCl/CFC. Compared to the previous chemical method [35,36,37], this method greatly shortens the electrode preparation time, saves energy and especially makes the electrochemical material pure. Moreover, the three electrodes are assembled on a polytetrafluoroethylene (PTFE) film simultaneously to make a flexible micro-sensor. The novel sensor possesses excellent performance in glucose sensing. Significantly, the sensor possesses flexible properties and shows ultrahigh sensitivity of 7.35 mA mM^−1^ cm^−2^ in the linear glucose concentration range of 0.10 to 3.30 mM. Meaningfully, the obtained device shows extreme reliability for practical glucose testing in human serum and juice samples, which makes it a good application prospect for flexible non-enzyme glucose sensors.

## 2. Materials and Methods

### 2.1. Reagents and Chemicals

Carbon fiber cloth (CFC) was purchased form Kunshan Tengerhui Electronic Technology Co., Ltd. (W0S1002). Copper (II) pyrophosphate (Cu_2_P_2_O_7_), potassium pyrophosphate (K_4_P_2_O_7_), ammonium citrate (C_6_H_17_N_3_O_7_), citric acid (C_6_H_8_O_7_), Na_2_SO_4_, HCl (36%) and KOH were purchased from Xilong Chemical Co., Ltd. (Guangzhou, China). Chloroauric acid (HAuCl_4_), chloroplatinic acid (H_2_PtCl_6_), potassium chloride (KCl), β-D-glucose (glucose), uric acid (UA, > 99%), dopamine (DA, > 98%), ascorbic acid (AA, > 99%), maltose, fructose, cysteine (Cys) and 4-acetamidophenol (4-AP) were received from Aladdin Company. AgNO_3_, Na_2_S_2_O_3_·5H_2_O, K_2_S_2_O_5_, NH_4_Ac and CH_5_N_3_S were purchased from Mclin Biochemical Technology Co., Ltd. All chemicals were of analytical grade without further purification. The human serum sample was obtained from Beijing Solarbio Science & Technology Co., Ltd. (Beijing, China) and the mulberry drink (Bosun) was purchased from a supermarket. Poly-tetra prone (PTFE) film and nano-double-sided tape were purchased from Jincheng Plastic Co., Ltd. and Qichen Office Supplies (Shenzhen) Co., Ltd., respectively.

### 2.2. Preparation of Cu/CFC

Firstly, carbon fiber cloth was immersed in 68% nitric acid at 90 °C for 30 min and then immersed into a 1 M KOH solution for 10 min to improve hydrophilic and remove organic binder on its surface. Then the processed carbon fiber cloth was stored in ethanol solution for later electrodeposition. Finally, the Cu film was electrodeposited on the processed carbon fiber cloth under 4 ASD (A dm^−2^) at 35 °C for 15 min and with a continuous stirring of 300 rpm, in which the copper plating solution was composed of 65 g L^−1^ Cu_2_P_2_O_7_, 380 g L^−1^ K_4_P_2_O_7_ and 22.5 g L^−1^ C_6_H_17_N_3_O_7_; the pH should be adjusted to 8.8 with citric acid. 

### 2.3. Preparation of Au@Cu(OH)_2_/CFC, Pt/CFC and Ag/AgCl/CFC

**Figure 1:**
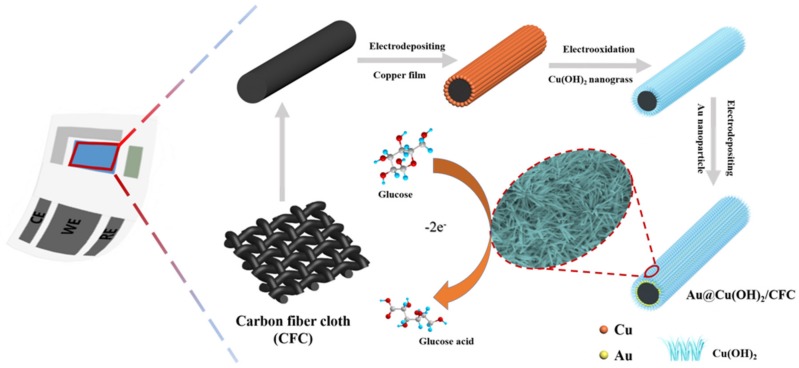
Schematic illustration of the fabrication process for the Au nanoparticle modified copper hydroxide nanograss arrays on flexible carbon fiber cloth (Au@Cu(OH)_2_/CFC) electrode and the corresponding detection mechanism.

The subsequent preparations were performed in a three-electrode system at 25 °C, in which commercial Pt foil and an Ag/AgCl (3 M KCl) electrode were used as counter electrode and reference electrode, respectively. After being washed with deionized water, the Cu/CFC electrode was immersed into 50 mL 1 M KOH solution and then immediately scanned three times with a linear sweep voltammetry (LSV) under the potential range from −0.40 to 0.40 V at 3 mV s^−1^ to oxidize the Cu on the CFC to Cu(OH)_2_. Then, the obtained Cu(OH)_2_/CFC electrode, after being rinsed with pure water, was put into a solution containing 1 mM HAuCl_4_ and 0.1 M KCl under a constant potential of −0.20 V for 180 s to electrodeposit Au nanoparticles to the surface of Cu(OH)_2_/CFC, that is, the Au@Cu(OH)_2_/CFC electrode was obtained. The synthesized processes were also illustrated in the scheme of Figure 1 and Figure S1 shows the corresponding optical images, from which it can be found that the color of the electrodes changes significantly at each stage (except the process of Au deposition). For comparison, an Au/CFC electrode was also prepared by employing the same electrodeposition procedure on CFC directly.

In our previous work, we fabricated a Pt counter electrode and an Ag/AgCl reference electrode [3,38]. In this paper, the same electrochemical method was used to fabricate the Pt/CFC and Ag/AgCl/CFC. It is worth noting that after open circuit potential testing the Ag/AgCl/CFC electrode can be fully usable (Figure S2, Appendix A).

The obtained Cu(OH)_2_/CFC, Au@Cu(OH)_2_/CFC, Pt/CFC and Ag/AgCl/CFC should be stored at 60 °C in a constant temperature dryer after washing with pure water and the subsequent drying procedure.

### 2.4. Preparation of the Flexible Integrated Sensor Device

As shown in Figure S3, the original blank electrodes used here are flexible carbon fiber cloth, including the working electrode with a size of 30 mm × 5 mm and leaving a 5 mm × 5 mm area to load the nanocomposites (Au@Cu(OH)_2_), the reference electrode with a size of 30 mm × 2.5 mm and leaving a 5 mm × 2.5 mm area to be loaded with Ag/AgCl and the counter electrode in a seven formation with a size of 32 mm × 2.5 mm as well as 9.5 mm × 2.5 mm and leaving 5 mm × 2.5 mm as well as a 5 mm × 2.5 mm area to be plated with Pt. After the three electrodes are fabricated, they are assembled on a poly-tetra prone (PTFE) film with a size of 40 mm × 20 mm at equal intervals of 2 mm. Then, another PTFE film with special openings (load material area of three electrodes) is laminated on it with nano-double-sided tape. In this way, a flexible micro-glucose sensor is obtained, the optical images of which can be seen in Figure S4.

### 2.5. Characterizations and Electrochemical Measurement

The surface morphologies of the electrodes were examined by Field Emission Scanning Electron Microscopy (SEM, Hitachi, SU8010, 10 keV) equipped with an energy dispersive spectrometer (EDS, IXRF). X-ray diffraction (XRD, Rigaku D-MAX 2200 VPC, Cu-Kα) was used to characterize the crystal structure of each sample. X-ray photoelectron spectroscopy (XPS) was collected with K-ALPHA^+^ spectroscopy using a monochromatic Al Kα X-ray source (1486.6 eV photons). Chronoamperometry (CA), linear sweep voltammetry (LSV), cyclic voltammetry (CV) and electrochemical impedance spectroscopy (EIS) were performed by Gamry Reference 600. In addition, all electrochemical tests were made in the three-electrode integrated system, in which the Au@Cu(OH)_2_/CFC, Cu(OH)_2_/CFC, Au/CFC and CFC electrodes prepared on the device were used as working electrodes, respectively. The as-prepared Pt electrode and Ag/AgCl/CFC electrode were, respectively, the counter electrode and reference electrode. It should be noted that in electrochemical tests, the area outside the working portion of the three electrodes is covered with Teflon tape to prevent the device from being damaged by the test solution. The volume of different solutions (with 0.01 M KCl inside to maintain the stability of the reference electrode) for all electrochemical tests are 50 mL and the components of each test solution were presented in the corresponding test section. In this paper all potentials were referenced to an as-prepared Ag/AgCl/CFC electrode and all LSV test potentials range from −0.3 V to 0.8 V unless otherwise stated. Besides this, all the CA tests were carried out with a continuous stirring of 200 rpm to allow the analyte to diffuse rapidly.

## 3. Results and Discussion

### 3.1. Characterizations of the Au@Cu(OH)_2_/CFC Sensor

**Figure 2:**
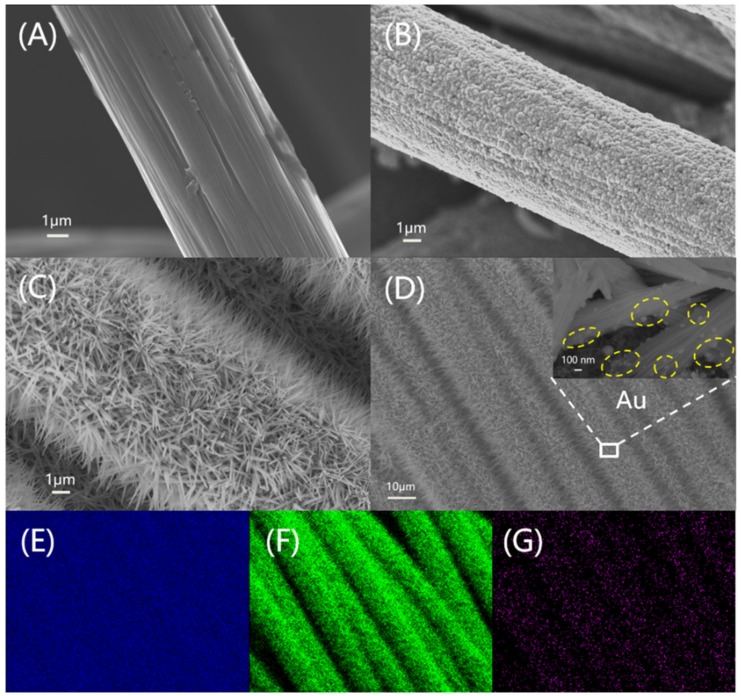
High-magnification SEM images of (**A**) CFC, (**B**) Cu/CFC and (**C**) Cu(OH)_2_/CFC, (**D**) Low-magnification SEM image of Au@Cu(OH)_2_/CFC (Inset is the partial enlarged view), EDX elemental mapping images of (**E**) Cu, (**F**) O and (**G**) Au corresponding to (**D**).

Figure 2A,B shows the SEM images of pre-treated carbon fiber cloth and Cu/CFC, respectively. Obviously, the copper film is plated on CFC successfully and the deposited copper is a layer of coarse copper particles, which can greatly increase the contact area between the copper film and the electrolyte solution. Cu(OH)_2_ arrays with a diameter about 100–280 nm and length of 1.5–2.0 μm show grass-like morphology on the surface of CFC (Figure 2C), which was converted from Cu/CFC by repeatedly scanning with LSV. The grass morphology structure significantly increased the space of Cu(OH)_2_/CFC and the possibility of providing more active sites [39]. Subsequently, Cu(OH)_2_/CFC was decorated with a tiny amount of Au nanoparticles by chronoamperometry (Figure 2D). Even after deposition of Au, the final Au@Cu(OH)_2_/CFC electrode still exhibits well-defined nanograss structure and the Au nanoparticles are distributed in the middle and lower parts of Cu(OH)_2_ nanograss, as seen from the inset of Figure 2D. As seen from Figure S5, both the obtained Pt/CFC and Ag/AgCl/CFC have a rough but very uniform structure.

To further ascertain the detailed composition and the crystal structure of the synthesized materials, EDS, XRD and XPS were performed. EDS element mapping images in Figure 2E–G demonstrate a uniform distribution of Cu, O and Au elements on the Au@Cu(OH)_2_/CFC. Figure S6A,B presents the typical EDS spectrums of Cu/CFC and Au@Cu(OH)_2_/CFC, with the core element compositions listed as insets. It is obvious that the atomic ratio of Cu to O elements is changed from about 22:5 (Figure S6A) to about 7:10 (Figure S6B), indicating a significant oxidation process on the Cu/CFC. Moreover, the Au element with an atom content of 4.30% (Figure S6B) verifies the presence of Au on the electrode surface. It is worth adding that the signal of C is mainly from the substrate (CFC).

**Figure 3:**
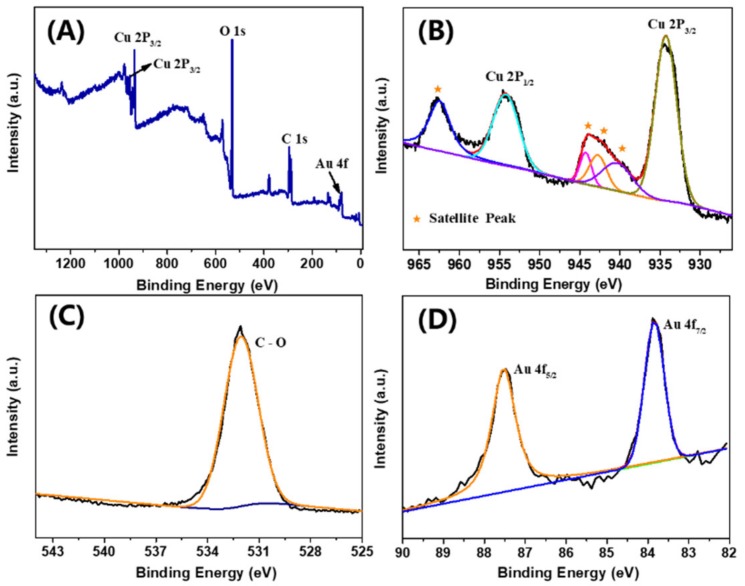
XPS spectra of Au@Cu(OH)_2_/CFC; (**A**) full spectrum survey; (**B**) Cu 2P; (**C**) O 1s; (**D**) Au 4f.

To avoid strong signal interference from the CFC substrate, a copper plate was chosen instead of it to be the substrate of Au@Cu(OH)_2_ for the XRD test. The XRD pattern of the Au@Cu(OH)_2_/Cu plate is recorded in Figure S6C, which is magnified in Figure S6D to interpret its plate pattern in a better manner. As shown in Figure S6D, there are seven diffraction peaks in the Au@Cu(OH)_2_/Cu plate at 16.7°, 23.8°, 34.1°, 35.8°, 38.0°, 39.8° and 53.2° corresponding to the (020), (021), (002), (011), (041), (130) and (150) crystal planes of Cu(OH)_2_ (JCPDS NO. 80-0656), respectively. The peaks at 38.2°, 44.5° and 64.7° correspond to the (111), (200) and (220) crystal planes of the cubic form of Au (JCPDS NO. 65-8601), respectively. Moreover, the three major peaks at 43.3°, 50.4° and 74.1° correspond to the (111), (200) and (220) crystal planes of Cu (JCPDS NO. 04-0836), respectively. To further confirm the material composition of the Au@Cu(OH)_2_/CFC, the XPS analysis was conducted. As shown in the full spectrum survey in Figure 3A, the Cu, O, C and Au elements exist in this sample. As shown in Figure 3B, the two peaks at 954.21 and 934.27 eV correspond to the Cu 2p_1/2_ and Cu 2p_3/2_ of the oxidized Cu(II) species, respectively, and the satellite peaks indicate the presence of high-spin Cu(II) ions [40]. It can be inferred that these Cu 2p peaks are consistent with Cu(OH)_2_ [41,42]. Figure 3C is the high-resolution XPS spectra of O 1s, in which a well-resolved O 1s peak is observed at 532.06 eV that further confirms the existence of Cu(OH)_2_ [43]. In addition, the binding energies of the doublet for Au 4f_7/2_ (83.84 eV) and Au 4f_5/2_ (87.48 eV) shown in Figure 3D are characteristic of Au (0) [44], which affirms that Au nanoparticles are decorated on the electrode as a pure element.

### 3.2. Mechanism of Electrode Fabrication and Glucose Oxidation

Figure S7A presents the LSV curves of fabricating Cu(OH)_2_/CFC electrodes. In the present conditions, the surface of the copper film is free of oxide at potentials negative of about -0.4 V and no Cu(III) species produce [45]. Obviously, there exist two anode peaks at around -112 mV (peak A) and 80 mV (peak B), which correspond to the oxidation processes of Cu to Cu(I) and Cu(I) to Cu(II), respectively [33,46]. The strength of both peaks drops sharply on the second and the third LSV curves, indicating that the conversions of copper to oxide are basically completed. The corresponding mechanism can be explained as follows:

(1) $$\mathrm{Peak}\;A:\;\mathrm{Cu}\;+\;{\mathrm{OH}}^{-}\;\to \;\mathrm{CuOH}\;+\;e^{-}$$

(2) $$2\;\mathrm{CuOH}\;↔\;{\mathrm{Cu}}_{2}O\;+\;H_{2}O$$

(3) $$\mathrm{Peak}\;B:\;\mathrm{CuOH}\;+\;{\mathrm{OH}}^{-}\;\to \;\mathrm{Cu}{\left(\mathrm{OH}\right)}_{2}\;+\;e^{-}$$

(4) $${\mathrm{Cu}}_{2}O\;+\;2\;{\mathrm{OH}}^{-}\;+\;H_{2}O\;\to \;2\;\mathrm{Cu}{\left(\mathrm{OH}\right)}_{2}\;+\;2e^{-}$$

**Figure 4:**
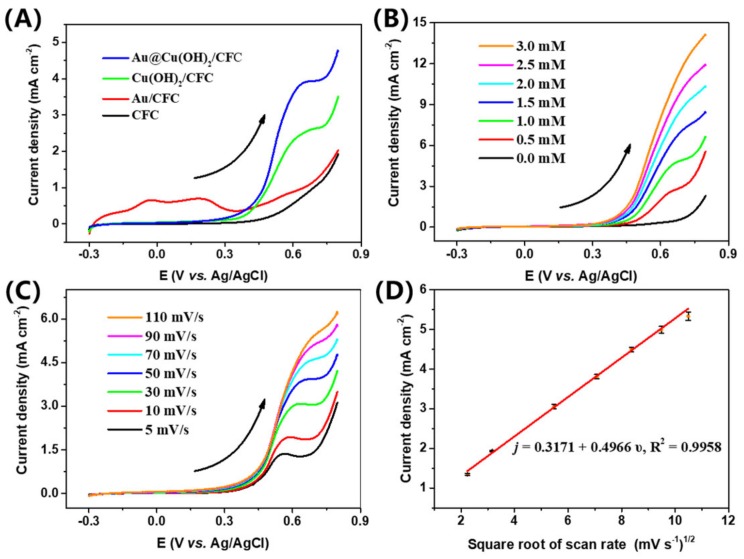
(**A**) Linear sweep voltammetry (LSV) curves of the bare CFC, Au/CFC, Cu(OH)_2_/CFC, Au@Cu(OH)_2_/CFC at a scan of 50 mV s^−1^ in 0.1 M KOH with 1.0 mM glucose. (**B**) LSV curves of Au@Cu(OH)_2_/CFC at different glucose concentrations (from 0.5 mM to 3.0 mM). (**C**) LSV curves of Au@Cu(OH)_2_/CFC at a scan rate of 5–110 mV s^−1^ in 0.1 M KOH with 1.0 mM glucose. (**D**) Relationship between square scan rate and the anodic peak currents, among which the maximum relative standard deviation (RSD) is 2.04%. The arrows along the LSV curves represent the direction of scanning.

The electrocatalytic activity of the composite material was first studied by the LSV. Figure S7B presents the LSV curves of the Cu(OH)_2_/CFC and Au@Cu(OH)_2_/CFC sensor devices in a 0.1 M KOH solution without and with 1 mM glucose, respectively. In the presence of glucose, a well-defined anodic peak appears at the potential of near 0.6 V for both Cu(OH)_2_/CFC and Au@Cu(OH)_2_/CFC devices, indicating an obvious process of glucose oxidation [47]. The LSV curves of Au@Cu(OH)_2_/CFC, Cu(OH)_2_/CFC, Au/CFC and CFC devices in 0.1 M KOH with 1 mM glucose are shown in Figure 4A; Cu(OH)_2_/CFC shows a strong response to glucose and has a distinct anodic peak, indicating the effective catalytic activity of Cu(OH)_2_. Similarly, Au@Cu(OH)_2_/CFC shows a stronger response starting from around 0.2 V. Moreover, there is an anodic peak in Au/CFC at around 0.5 V, consistent with our previous study that nanoporous Au catalyzes glucose in the around range of 0.2 to 0.52 V [38]. In order to further study the role of gold nanoparticles in nanocomposites, the electrochemically active surface areas (ECSA) and the impedances of the composite electrodes were measured.

ECSA proportional to the double layer capacitance (*C_dl_*) is usually used to evaluate the active sites of the electrocatalyst [48,49]. Herein, the *C_dl_* was obtained by the CV method (Figure S8A,B) in 0.1 M KOH at various scan rates (10–50 mV s^−1^) with the corresponding cyclic voltammograms −0.20 to −0.10 V where the current changes are due to the charges of the double layer [50]. Figure S8C shows the linearly fitted relationships between *Δj*_−0.15V_ and *v* of Cu(OH)_2_/CFC and Au@Cu(OH)_2_/CFC. Obviously, the *C_dl_* of Au@Cu(OH)_2_/CFC (1.0804 mF cm^−2^) is much larger than that of Cu(OH)_2_/CFC (0.4339 mF cm^−2^), which suggests the higher ECSA of Au@Cu(OH)_2_/CFC and further demonstrates that the Au nanoparticles greatly help increasing the catalytic active sites [48,50]. Figure S8D shows the Nyquist plots of Cu(OH)_2_/CFC and Au@Cu(OH)_2_/CFC electrodes in the frequency range from 10^−1^ to 10^5^ Hz and the measurement was conducted in 5 mM K_3_[Fe(CN)_6_]/K_4_[Fe(CN)_6_] with a 0.1 M KCl supporting electrolyte. It can be found that both Nyquist plots consist of a semicircle portion at high frequencies and a linear portion at low frequency ranges that correspond to the electron-transfer limited and diffusion limited processes, respectively [51]. The inset represents the equivalent Randle circuit for the two cases and the Rct (charge-transfer resistance) of the Au@Cu(OH)_2_/CFC (Rct = 12.03 Ω cm^2^) is found to be much lower than that of the Cu(OH)_2_/CFC (Rct = 27.72 Ω cm^2^). This indicates that the modification of Au can significantly enhance the charge transfer ability of the electrode during the electrocatalytic process [52].

Above all, the electrocatalytic process for glucose oxidation on the Au@Cu(OH)_2_/CFC electrode is mediated by the Cu(II)/Cu(III) and Au(0)/Au(I) redox couples, as expressed in Equations (5)–(8) [31,38,53]. During anodic scan in an alkaline medium, the Cu(OH)_2_ and Au are first electrooxidized to CuOOH and Au(OH)_ads_ active intermediates. Then they could oxidize glucose to gluconic acid after the addition of glucose.

(5) $$\mathrm{Cu}(\mathrm{OH}{)}_{2}\;+\;{\mathrm{OH}}^{-}\;\to \;\mathrm{CuOOH}\;+\;e^{-}\;+\;H_{2}O$$

(6) $$\mathrm{Au}\;+\;{\mathrm{OH}}^{-}\;\to \;\mathrm{Au}{\left(\mathrm{OH}\right)}_{\mathrm{ads}}\;+\;e^{-}$$

(7) $$2\;\mathrm{CuOOH}\;+\;C_{6}H_{12}O_{6}\;(\mathrm{Glucose})\;+\;H_{2}O\;\to \;C_{6}H_{12}O_{7}\;(\mathrm{Glucose}\;\mathrm{acid})\;+\;2\;\mathrm{Cu}(\mathrm{OH}{)}_{2}$$

(8) $$2\;\mathrm{Au}{\left(\mathrm{OH}\right)}_{\mathrm{ads}}\;+\;C_{6}H_{12}O_{6}\;(\mathrm{Glucose})\;\to \;C_{6}H_{12}O_{7}\;(\mathrm{Glucose}\mathrm{acid})\;+\;2\;\mathrm{Au}\;+\;H_{2}O$$

### 3.3. Non-Enzymatic Glucose Electrochemical Performance of an Au@Cu(OH)_2_/CFC Sensor

Figure 4B displays the LSV curves of the Au@Cu(OH)_2_/CFC in 0.1 M KOH with different concentrations of glucose (ranging from 0 to 3.0 mM) at a scan rate of 50 mV s^−1^. The response current enhances obviously following the increase of the glucose concentration, which indicates a good catalytic effect of Au@Cu(OH)_2_/CFC towards glucose [54]. The LSV curves of Au@Cu(OH)_2_/CFC in 0.1 M KOH with 1 mM glucose at different scan rates (from 5 to 110 mV s^−1^) are depicted in Figure 4C. Obviously, the anodic peak current increases as the scan rates increases. In addition, the oxidation peak current density *j* (mA cm^−2^) against square root of scan rate ν (mV s^−1^) shows a brilliant linear relationship (Figure 4D) which can be expressed as: *j* = 0.4966*ν^1/2^+ 0.3171 (R^2^ = 0.9958). The relationship suggests that the electrochemical behavior of glucose oxidation on Au@Cu(OH)_2_/CFC is controlled by a diffusion process.

As can be seen from Equations (5) and (6), the OH^−^ plays an important role in the catalytic oxidation of glucose. Therefore, different concentrations of KOH solutions (ranging from 0.01 to 1.00 M) were researched by CA tests with the addition of 0.1 mM glucose at 0.60 V to optimize the sensing performance of the Au@Cu(OH)_2_/CFC (Figure S9A). The results demonstrate that the optimal concentration of KOH is 0.1 M. After a similar screening experiment, the optimized test potential for the CA tests is determined to be 0.60 V (Figure S9B). Hence, a 0.10 M KOH solution and 0.60 V are selected as the optimal electrolyte and test potential, respectively, for all the subsequent CA tests.

**Figure 5:**
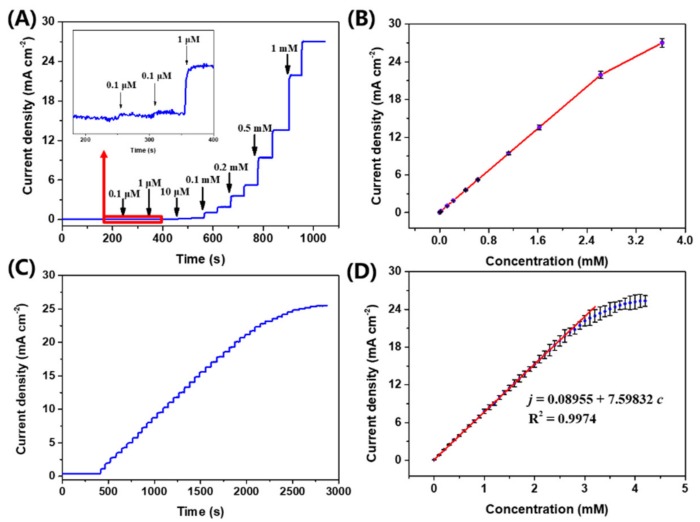
(**A**) Chronoamperometric response of Au@Cu(OH)_2_/CFC towards the successive additions of different concentrations of glucose. (**B**) The corresponding calibration curve of Figure A. (**C**) Chronoamperometric response of Au@Cu(OH)_2_/CFC towards the successive additions of 0.1 mM glucose. (**D**) The calibration curve for chronoamperometric response to glucose concentration in the high concentration range (corresponding to Figure C), among which the maximum RSD is 5.1%.

Figure 5A presents the chronoamperometric response curve of an Au@Cu(OH)_2_/CFC sensor towards the successive addition of glucose. For ease of observation, the current response curve of Au@Cu(OH)_2_/CFC to low concentrations of glucose is inserted in Figure 5A. It can be easily found that an Au@Cu(OH)_2_/CFC sensor device has a current response sensitive to glucose even diluted to 0.1 μM, and the current response exhibits a linear relationship towards the addition of glucose (Figure 5B). In order to explore the exact upper limit of the linear range, the chronoamperometric response of Au@Cu(OH)_2_/CFC towards the successive additions of 0.1 mM glucose was measured (Figure 5C). As shown in Figure 5D, the current response is linearly enhanced up to 3.3 mM; after that, the enhancement of the current response is gradually weakened. The linear relationship between the current density *j* (mA cm^−2^) and the corresponding glucose concentration *c* (mM) can be calculated by the equation: *j* = 7.5983 *c* + 0.0896 with correlation coefficients of 0.9974. Therefore, the sensitivity of the Au@Cu(OH)_2_/CFC electrode towards glucose detection is 7.5983 mA mM^−1^ cm^−2^ and the limit of detection (LOD) can be calculated to be 26.97 nM (S/N = 3) by the criteria formula of LOD = 3$\delta_{b}$/*m,* where $\delta_{b}$ is the standard deviation (7.15 × 10^−5^ mA cm^−2^) of the blank sample and *m* is the sensitivity of the electrode [51].

Table S1 lists the glucose catalytic performances of the Au@Cu(OH)_2_/CFC sensor device and some other composites materials. Obviously, compared to the commonly used non-electrochemical methods for detecting glucose, the Au@Cu(OH)_2_/CFC sensor has the advantages of better sensitivity, faster response and being more economical. Furthermore, our device also shows much better sensitivity and a lower detection limit in comparison to these similar electrocatalyst materials. Thus, it is a promising candidate for the application of the three-electrode integrated device structure and Au@Cu(OH)_2_/CFC nanocomposite on glucose detection.

### 3.4. Selectivity, Stability and Reproducibility of the Au@Cu(OH)_2_/CFC Sensor

**Figure 6:**
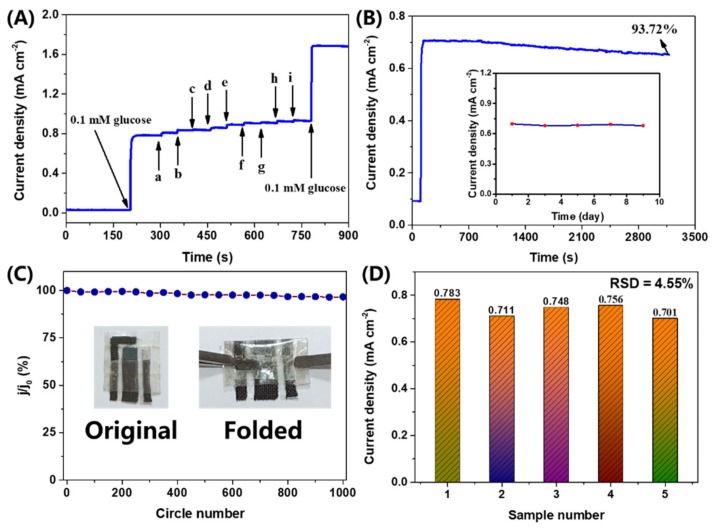
(**A**) Chronoamperometric response of Au@Cu(OH)_2_/CFC sensor toward the addition of 0.1 mM glucose and various interfering species. (a–i are Maltose, Fructose, UA, AA, DA, Cys, 4-AP, KCl and Na_2_SO_4_, respectively). (**B**) Long-term stability tests of the Au@Cu(OH)_2_/CFC sensor in 0.1 M KOH and 0.01 M KCl solution containing 0.1 mM glucose for over 3000 s and 9 days (inset). (**C**) Folded stability test of the Au@Cu(OH)_2_/CFC sensor; inset shows the repeating folded and recovery process (j is current response density of the original sensor and j_0_ is current response density of the folded sensor). (**D**) The chronoamperometric current response of five pieces Au@Cu(OH)_2_/CFC towards 0.1 mM glucose.

Selectivity is also a significant indicator of Au@Cu(OH)_2_/CFC for glucose detection. Hence, various conventional organic and inorganic ions were selected for an anti-interference test of Au@Cu(OH)_2_/CFC for glucose detection. The general glucose concentration in humans is fairly higher than that of the coexisting interferences such as maltose, fructose, UA, DA, AA, Cys and 4-AP [15], so the concentration of each interference (0.01 mM) was selected to be one tenth of the glucose concentration (0.1 mM) for the anti-interference test (Figure 6A). In addition, high concentrations (1.00 mM) of KCl and Na_2_SO_4_ were added to determine the anti-interference ability of Au@Cu(OH)_2_/CFC to conventional inorganic ions. As the results show in Table S2, the signals of organic interferences are less than 4%, and the response signal to inorganic substances is smaller (less to 2%). This test demonstrates that the Au@Cu(OH)_2_/CFC is highly selective for glucose detection and is almost unaffected by conventional inorganic ions (K^+^, Na^+^, Cl^−^). Figure 6B presents the continuous chronoamperometric response of Au@Cu(OH)_2_/CFC towards 0.1 M glucose to measure the long-term operation ability of the electrode for glucose detection. The result expounds that Au@Cu(OH)_2_/CFC can keep a stable current response for over 3000 s with only 6.28% decay. Moreover, the same Au@Cu(OH)_2_/CFC sensor was tested in the same concentration of glucose solution at the same time every two days and it could keep with a stable performance for nine days, as demonstrated in the inset of Figure 6B. Thus, our Au@Cu(OH)_2_/CFC glucose sensor has potential in practical applications with stable performance. The folded stability of the flexible device was further tested. As presented in Figure 6C, it shows nearly no change in the response to glucose after 1000 folded cycles, demonstrating the excellent flexible capacity of the obtained sensor. Five pieces of the Au@Cu(OH)_2_/CFC sensor were fabricated using the same preparation process and their chronoamperometric responses towards 0.1 mM glucose are collected in Figure S6D to study the reproducibility. The low relative standard deviation (RSD) of 4.55% firmly certifies the reliability of the preparation process.

### 3.5. Real Sample Analysis

To evaluate the capability of the Au@Cu(OH)_2_/CFC sensor for practical determination, the device was used to detect the glucose concentrations of human serum samples and mulberry drink by the standard addition method [55]. Before the test, the mulberry drink was centrifuged at 5000 rpm for 30 min and then the serum and mulberry juice were separately diluted 500-fold and 50,000-fold with 0.1 M KOH to reduce the matrix effect [56]. Finally, the glucose concentration of human serum, mulberry drink and the spiked samples (the mulberry drink samples with added known concentrations of glucose) were measured. From the results listed in Table 1, good recoveries from 92.09% to 97.89% with low RSDs (0.75% to 1.02%) are achieved for mulberry juice detection. Besides this, the glucose concentration of human serum samples measured by an Au@Cu(OH)_2_/CFC sensor is consistent with the values detected by a blood glucose meter (SANNUO GA-3, Sinocare Inc., Changsha). These results indicate that the Au@Cu(OH)_2_/CFC sensor is excellently reliable in actual glucose detection.

## 4. Conclusions

In this paper, the flexible Au@Cu(OH)_2_/CFC, Pt/CFC and Ag/AgCl/CFC electrodes were successfully synthesized by simple electrochemical deposition and oxidation methods. And a flexible three-electrode integrated portable glucose sensor was successfully fabricated, which consists of Au@Cu(OH)_2_/CFC as the working electrode, Pt/CFC as the counter electrode and Ag/AgCl/CFC as the reference electrode. Due to the following reasons, (I) Cu(OH)_2_ nanograss arrays possess superior electrochemical catalytic activity for glucose sensing; (II) the modification of the Au nanoparticles accelerates the charge transfer and increases the electrochemically active surface area of the nanograss arrays, providing more catalytic active sites during the electrocatalytic process in an alkaline environment; (III) intimate contact between the active material (Au@Cu(OH)_2_) and current collector (CFC) with a macroporous structure supplies good conductivity for electron/ion transport, the sensor exhibited very high sensitivity of 7.35 mA mM^−1^ cm^−2^, low limit of detection of 26.97 nM (S/N = 3), superb selectivity and considerable stability towards glucose detection. Additionally, the Au@Cu(OH)_2_/CFC flexible portable sensor shows excellent flexible capacity and good reliability in actual glucose detection. All in all, the novel sensor can provide a promising structure design for a flexible micro-device and is a great potential material candidate of an electrochemical sensor for glucose sensing.

## Figures and Tables

**Table 1:** The detection of glucose concentrations in the human serum sample and mulberry drink.

Samples	Reference Values (mM)	Added Glucose (mM)	Au@Cu(OH)_2_/CFC^c^ (mM)	Recovery (%)	RSD (%, n = 3)
Mulberry juice	670.68^a^	400.00	1030.83 ± 8.22	92.09	0.75
		800.00	1439.60 ± 15.23	97.89	1.02
Human serum	5.4^b^	-	5.37	99.44	0.81

^a^ Measured by standard addition method, ^b^ measured by blood glucose meter (GA-3), ^c^ expressed as “mean value ± standard deviation”.

## Supplementary Materials

The following are available online at https://www.mdpi.com/1424-8220/19/22/5055/s1, Figure S1: Optical images of (A) CFC, (B) Cu/CFC, (C) Cu(OH)2/CFC (D) Au@Cu(OH)2/CFC, Figure S2: Open circuit potential (OCP) between deposited Ag/AgCl/CFC electrode and a commercial Ag/AgCl (3 M KCl) reference electrode in electrolyte solution with 0.1 M KOH and 0.01 M KCl, Figure S3: Schematic illustration of the fabrication process for the flexible micro glucose sensor, Figure S4: Optical images of the (A) original and (B) folded flexible micro glucose sensor, Figure S5: Low-magnification SEM images of (A) Ag/AgCl/CFC and (B) Pt/CFC, and High-magnification SEM images of (C) Ag/AgCl/CFC and (D) Pt/CFC, Figure S6: EDS patterns of (A) Cu/CFC and (B) Au@Cu(OH)2/CFC, XRD patterns of (C) Au@Cu(OH)2/Cu plate and (D) The magnification of (C), Figure S7: LSV curves of (A) fabricating Cu(OH)2 samples, from −0.4 V to 0.4 V with 3 mV s−1 sweeping rate in 1 M KOH resolution for three times, (B) the Cu(OH)2/CFC and Au@Cu(OH)2/CFC sensors in 0.1 M KOH and 0.01 M KCl solution without and with 1 mM glucose, Figure S8: CVs for (A) Cu(OH)2/CFC and (B) Au@Cu(OH)2/CFC sensors with different scan rates( 10–50 mV s−1) in 0.1 M KOH and 0.01 M KCl solution, (C) The capacitive current densities-scan rates calibration plots of the two sensors at −0.15 V (Δj−0.15V = (ja − jc)/2), (D) Nyquist plots of Cu(OH)2/CFC and Au@Cu(OH)2/CFC sensors in 0.1 M KCl electrolyte with 5 mM K3[Fe(CN)6] and 5 mM K4[Fe(CN)6], inset is equivalent Randle circuit for the two cases, Figure S9: (A) The line chart of chronoamperometric current response of Au@Cu(OH)2/CFC towards 0.10 mM glucose in different concentration KOH (0.01 to 1.00 M) and 0.01 M KCl solution at 0.60V. The maximum RSD is 2.8%. (B) The chronoamperometric current response of Au@Cu( OH)2/CFC towards 0.10 mM glucose in 0.1 M KOH and 0.01 M KCl solution at different potentials (ranging from 0.30 to 0.65 V), Table S1: The comparison of glucose sensing performances based on various composites which reported previously. SPE: screen printed electrode, GCE: glassy carbon electrode, rGO: reduced graphene oxide, PGF: porous graphene foam, MWCNTs: multi-walled carbon nanotubes array, Table S2: Table S2. Influence of common interfering species on the determination of glucose with Au@Cu(OH)2/CFC in 0.1 M KOH and 0.01 M KCl solution.

## Appendix A

A detailed description of the open circuit potential test mentioned in Section 2.3.

According to the Nernst equation, the relationship between *E_Ag/AgCl_* and chloride ions activity *A_Cl_^−^* (25 °C) can be described as the equation: *E_Ag/AgCl_* (mV) *=* 222 − 59 lg*A_Cl_^−^*. Generally, *A_Cl_^−^* was replaced by chloride ion concentration *C_Cl_^−^* for approximate calculation. Thus, the theoretical value of the electric potential difference between the prepared Ag/AgCl/CFC (0.01 M KCl) and the commercial Ag/AgCl (3 M KCl) reference electrode was calculated to be 146 mV, which could be described as the equation: *E_Ag/AgCl_* (3 M KCl, mV) = *E_Ag/AgCl_* (0.01 M KCl) − 146. In order to study the availability of the prepared Ag/AgCl/CFC, an open circuit potential (OCP) test was performed in a two-electrode system, in which the obtained Ag/AgCl/CFC worked as the working electrode and the commercial Ag/AgCl (3 M KCl) reference electrode worked as the counter and reference electrode. As seen from Figure S2, the OCP finally kept steady at 141.6 mV with less than 0.5 mV variation. This result is consistent with the theoretical value, which strongly confirms the availability of the prepared Ag/AgCl/CFC reference electrode.
