# Supplementary material for: A Flexible Portable Glucose Sensor Based on Hierarchical Arrays of Au@Cu(OH)2 Nanograss
Source: Sensors (Basel). 2019 Nov 19;19(22):5055. doi: 10.3390/s19225055 (PMC6891777; doi:10.3390/s19225055)
Supplement: Supplementary file 1 [file sensors-19-05055-s001.pdf]

A Flexible Portable Glucose Sensor Based on Hierarchical Arrays of Au@Cu(OH)₂ Nanograss

Min Jiang [1], Peng Sun [2,3], Jie Zhao [1,*], Lihua Huo [4,*] and Guofeng Cui [2,3,*]

[1] School of Mechanical and Automotive Engineering, South China University of Technology, Guangzhou, 510640, China; mejiangmin@mail.scut.edu.cn

[2] Key Laboratory for Polymeric Composite & Functional Materials of Ministry of Education, School of Chemistry, Sun Yat-sen University, Guangzhou, 510275, China; sunp25@mail2.sysu.edu.cn

[3] MOE Laboratory of Bioinorganic and Synthetic Chemistry, The Key Lab of Low-Carbon Chemistry and Energy Conservation of Guangdong Province, School of Chemistry, Sun Yat-sen University, 135, Xingang West Road, Guangzhou, 510275, China

[4] Key Laboratory of Functional Inorganic Material Chemistry, Ministry of Education, School of Chemistry and Materials Science, Heilongjiang University, Harbin 150080, China

* Correspondence: zhaoj77@scut.edu.cn (J.Z.); huolihua@hlju.edu.cn (L.H.); cuigf@mail.sysu.edu.cn (G.C.)

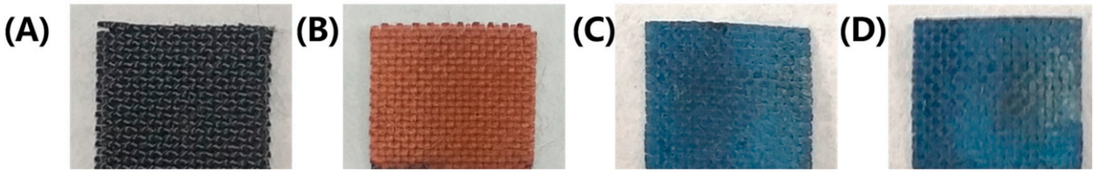

Figure S1. Optical images of (**A**) CFC, (**B**) Cu/CFC, (**C**) $Cu(OH)_2$/CFC (**D**) Au@$Cu(OH)_2$/CFC.

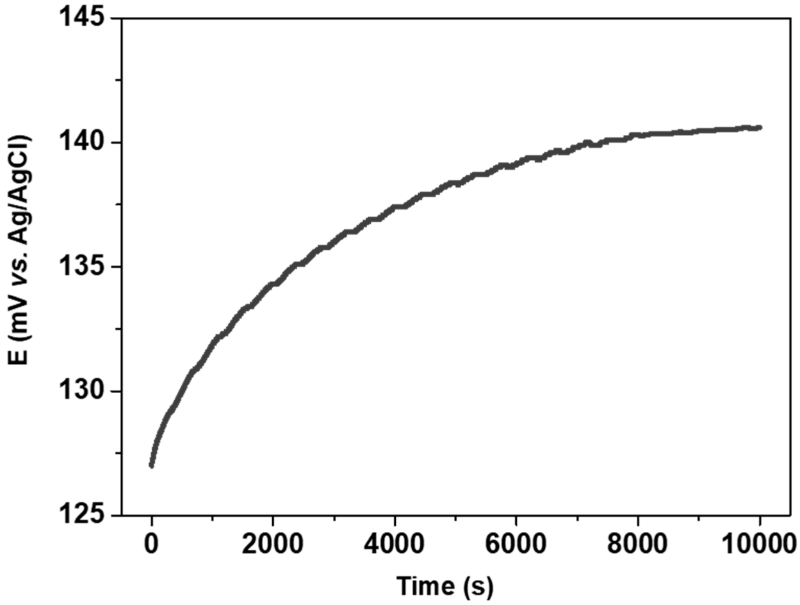

Figure S2. Open circuit potential (OCP) between deposited Ag/AgCl/CFC electrode and a commercial Ag/AgCl (3 M KCl) reference electrode in electrolyte solution with 0.1 M KOH and 0.01 M KCl.

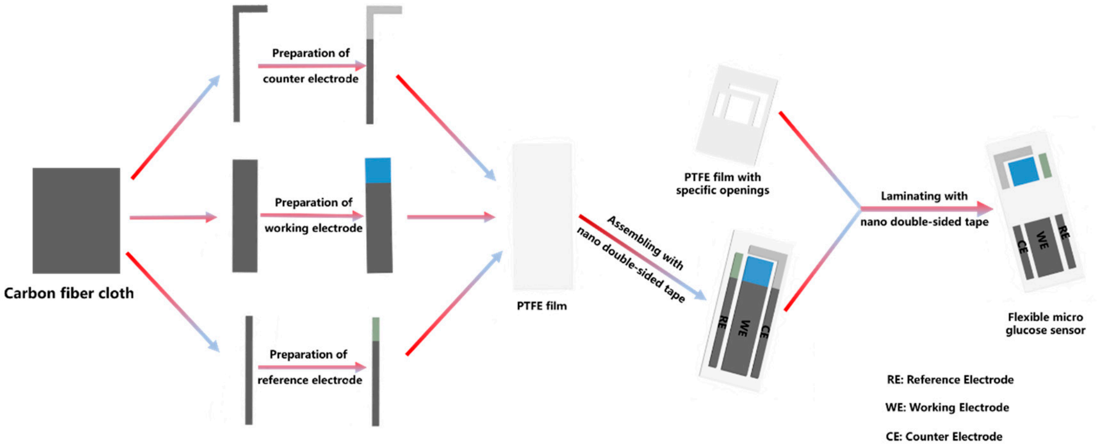

Figure S3. Schematic illustration of the fabrication process for the flexible micro glucose sensor.

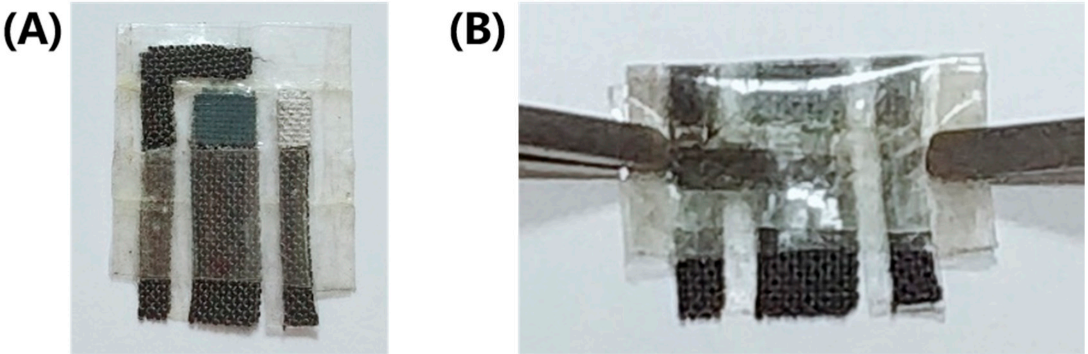

Figure S4. Optical images of the (**A**) original and (**B**) folded flexible micro glucose sensor.

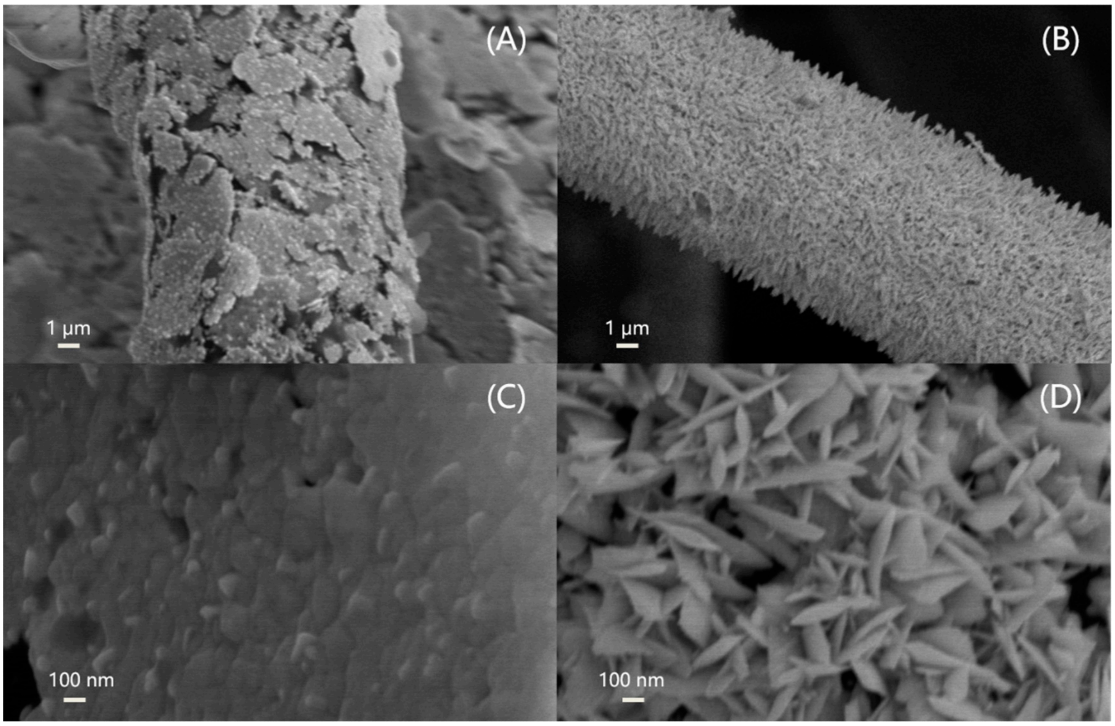

Figure S5. Low-magnification SEM images of (**A**) Ag/AgCl/CFC and (**B**) Pt/CFC, and High-magnification SEM images of (**C**) Ag/AgCl/CFC and (**D**) Pt/CFC.

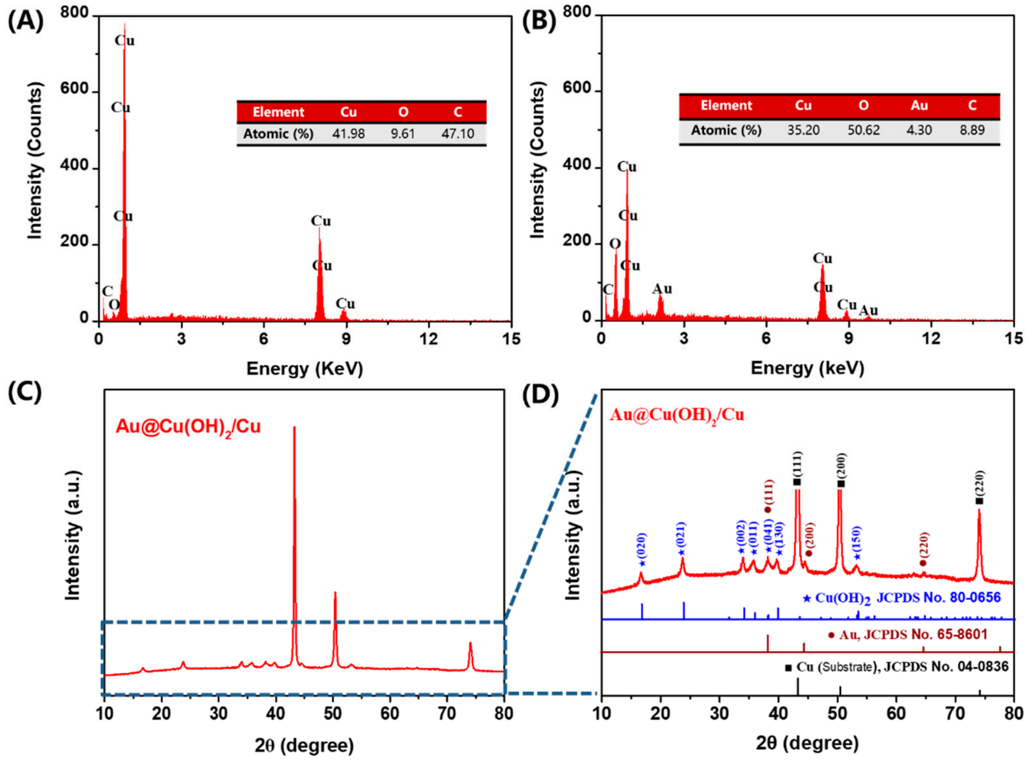

Figure S6. EDS patterns of (**A**) Cu/CFC and (**B**) Au@$Cu(OH)_2$/CFC, XRD patterns of (**C**) Au@$Cu(OH)_2$/Cu plate and (**D**) The magnification of (C).

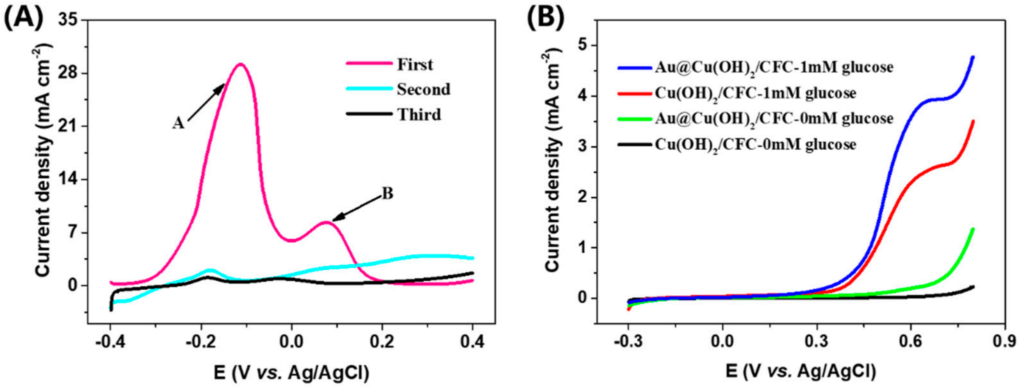

Figure S7. LSV curves of (**A**) fabricating $Cu(OH)_2$ samples, from −0.4 V to 0.4 V with 3 mV s^{-1} sweeping rate in 1 M KOH resolution for three times, (**B**) the $Cu(OH)_2$/CFC and Au@$Cu(OH)_2$/CFC sensors in 0.1 M KOH and 0.01 M KCl solution without and with 1 mM glucose.

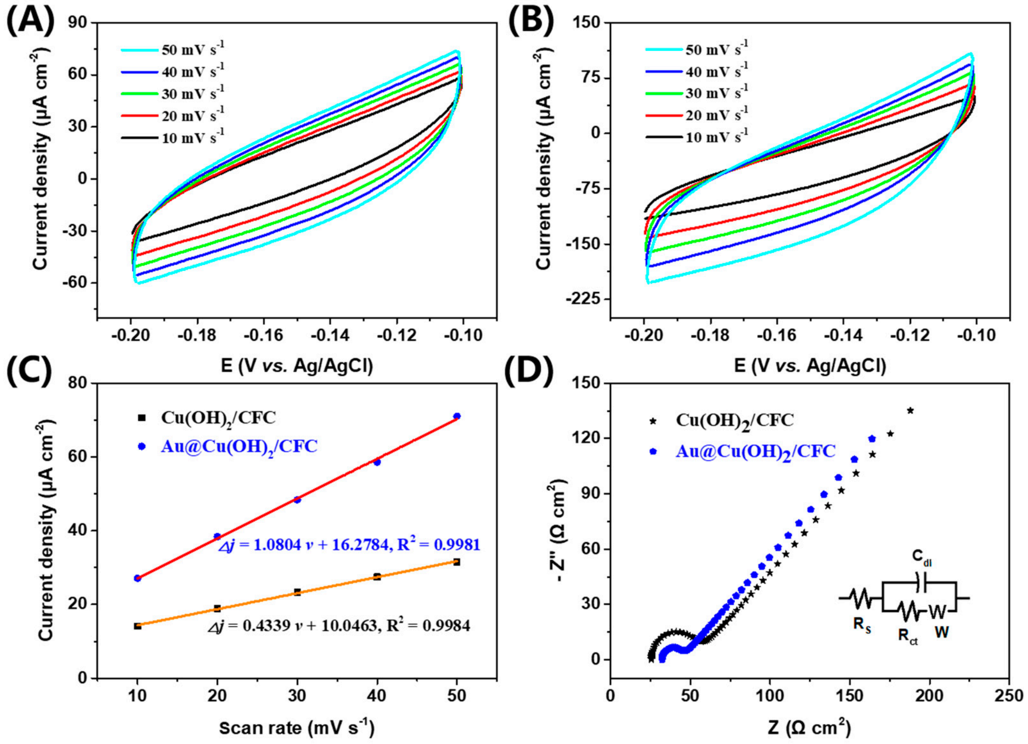

Figure S8. CVs for (A) $Cu(OH)_2$/CFC and (B) Au@$Cu(OH)_2$/CFC sensors with different scan rates(10–50 mV s^{-1}) in 0.1 M KOH and 0.01 M KCl solution, (C) The capacitive current densities-scan rates calibration plots of the two sensors at −0.15 V ($\Delta j_{-0.15V} = (j_a - j_c)/2$), (D) Nyquist plots of $Cu(OH)_2$/CFC and Au@$Cu(OH)_2$/CFC sensors in 0.1 M KCl electrolyte with 5 mM $K_3[Fe(CN)_6]$ and 5 mM $K_4[Fe(CN)_6]$, inset is equivalent Randle circuit for the two cases.

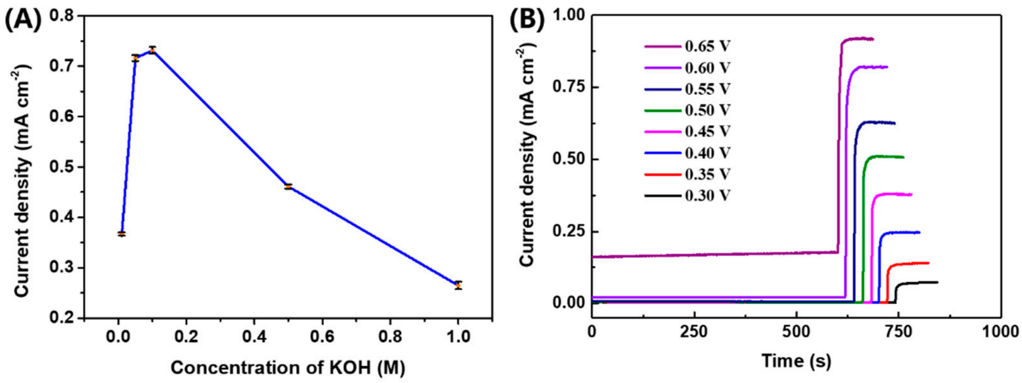

Figure S9. (A) The line chart of chronoamperometric current response of Au@$Cu(OH)_2$/CFC towards 0.10 mM glucose in different concentration KOH (0.01 to 1.00 M) and 0.01 M KCl solution at 0.60V. The maximum RSD is 2.8%. (B) The chronoamperometric current response of Au@Cu($OH)_2$/CFC towards 0.10 mM glucose in 0.1 M KOH and 0.01 M KCl solution at different potentials (ranging from 0.30 to 0.65 V).

Table S1. The comparison of glucose sensing performances based on various composites which reported previously. SPE: screen printed electrode, GCE: glassy carbon electrode, rGO: reduced graphene oxide, PGF: porous graphene foam, MWCNTs: multi-walled carbon nanotubes array.

Electrode	Detection Method	Response time (s)	Sensitivity	Detection limit (μM)	Linear range (mM)	Cost	Refs
GQDs	Optical	~	0.012*	3000	4–40	Very high	[1]
polymer optode	Optical	900 ~	0.2*	1000	1–19	Very high	[2]
nanoporous pigments	Colorimetric	30	-	1000	2–50	high	[3]
Polymer Gel	Colorimetric	10^4 ~	-	5000	5–8	high	[4]
Au-CuO/rGO/SPE	LSV	10	2.36#	0.100	0.0001–12	low	[5]
CuO/GCE-Nafion	Amperometry	5	0.47#	0.016	1–10	low	[6]
Cu_2O/graphene nanosheets	Amperometry	5	0.29	3.300	0.3–3.3	low	[7]
$Cu(OH)_2$ nanotubes	Amperometry	5	0.42#	0.500	up to 3	low	[8]
CuO/rGO/GCE	Amperometry	5	1.36#	0.700	0.002–4	low	[9]
CuO/carbon spheres	Amperometry	5	2.98#	0.100	0.0005–2.3	low	[10]
CuO-MWCNTs	Amperometry	2	2.19#	0.800	0.2–3	low	[11]
$Cu(OH)_2$/PGF	Amperometry	10	3.36#	1.200	0.00012–6	low	[12]
CuO arrays/ nanoporous Cu	Amperometry	3	1.62#	0.200	0.0005–5.0	low	[13]
Au@$Cu(OH)_2$/CFC	Amperometry	5	7.35#	0.027	0.1–3.3	low	This work

* mM per percent change of fluorescence intensity, # mA mM^{-1} cm^{-2}.

Table S2. Influence of common interfering species on the determination of glucose with Au@$Cu(OH)_2$/CFC in 0.1 M KOH and 0.01 M KCl solution.

Label	Analyte	Tested Concentration(mM)	Current Response (%)
-	Glucose	0.1	100
a	Maltose	0.01	3.46
b	Fructose	0.01	3.79
c	Uric acid (UA)	0.01	1.13
d	Dopamine (DA)	0.01	1.93
e	Ascorbic acid (AA)	0.01	3.83
f	Cysteine	0.01	2.29
g	Acetaminophen	0.01	0.04
h	KCl	1	1.80
i	Na_2SO_4	1	1.56

References

1. Shehab, M.; Ebrahim, S.; Soliman, M. Graphene quantum dots prepared from glucose as optical sensor for glucose. *J Lumin* **2017**, *184*, 110–116.
2. Billingsley, K.; Balaconis, M.K.; Dubach, J.M.; Zhang, N.; Lim, E.; Francis, K.P.; Clark, H.A.; Ester, G.B. Fluorescent Nano-Optodes for Glucose Detection. *Anal Chem* **2010**, *82*, 3707–3713.
3. Lim, S.H.; Musto, C.J.; Park, E.; Zhong, W.; Suslick, K.S. A Colorimetric. Sensor Array for Detection and Identification of Sugars. *Org Lett* **2009**, *10*, 4405–4408.
4. Honda, M.; Kataoka, K.; Seki, T.; Takeoka, Y. Confined Stimuli-Responsive Polymer Gel in Inverse Opal Polymer Membrane for Colorimetric Glucose Sensor. *Langmuir* **2009**, *25*, 8349–8356.
5. Dhara, K.; Ramachandran, T.; Nair, B.G.; Satheesh Babu, T.G. Single step synthesis of Au-CuO nanoparticles decorated reduced graphene oxide for high performance disposable nonenzymatic glucose sensor. *J Electroanal Chem* **2015**, *743*, 1–9.
6. Baloach, Q.A.; Tahira, A.; Mallah, A.B.; Abro, M.I.; Uddin, S.; Willander, M.; Ibupoto, Z.H. A robust, enzyme-free glucose sensor based on lysine-assisted CuO nanostructures. *Sensors* **2016**, *16, 1878–1888.*
7. Liu, M.; Liu, R.; Chen, W. Graphene wrapped Cu_2O nanocubes: Non-enzymatic electrochemical sensors for the detection of glucose and hydrogen peroxide with enhanced stability. *Biosens Bioelectron* **2013**, *45*, 206–212.
8. Zhou, S.; Feng, X.; Shi, H.; Chen, J.; Zhang, F.; Song, W. Direct growth of vertically aligned arrays of $Cu(OH)_2$ nanotubes for the electrochemical sensing of glucose. *Sensors Actuators B Chem* **2013**, *177*, 445–452.
9. Luo, L.; Zhu, L.; Wang, Z. Nonenzymatic amperometric determination of glucose by CuO nanocubes – graphene nanocomposite modified electrode. *Bioelectrochemistry* **2012**, *88*, 156–163.
10. Zhang, J.; Ma, J.; Zhang, S.; Wang, W.; Chen, Z. A highly sensitive nonenzymatic glucose sensor based on CuO nanoparticles decorated carbon spheres. *Sensors Actuators B Chem* **2015**, *211*, 385–391.
11. Yang, J.; Jiang, L.; Zhang, W.; Gunasekaran, S. A highly sensitive non-enzymatic glucose sensor based on a simple two-step electrodeposition of cupric oxide (CuO) nanoparticles onto multi-walled carbon nanotube arrays. *Talanta* **2010**, *82*, 25–33.
12. Shackery, I.; Patil, U.; Pezeshki, A.; Shinde, N.M.; Kang, S.; Im, S.; Chan, S. Copper Hydroxide Nanorods Decorated Porous Graphene Foam Electrodes for Non-enzymatic Glucose Sensing. *Electrochim Acta* **2016**, *191*, 954–961.
13. Chen, H.; Fan, G.; Zhao, J.; Qiu, M.; Sun, P.; Fu, Y.; Han, D.; Cui, G. A portable micro glucose sensor based on copper-based nanocomposite structure. *New J Chem* **2019,** 43, 7806–7813.
